# Development of an Innovative in Vitro Potency Assay for Anti-Botulinum Antitoxins

**DOI:** 10.3390/toxins8100276

**Published:** 2016-09-24

**Authors:** Osnat Rosen, Eyal Ozeri, Ada Barnea, Alon Ben David, Ran Zichel

**Affiliations:** Department of Biotechnology, Israel Institute for Biological Research, Ness Ziona 7410001, Israel; osnatr@iibr.gov.il (O.R.); eyalo@iibr.gov.il (E.O.); adab@iibr.gov.il (A.B.); alonb@iibr.gov.il (A.B.D.)

**Keywords:** botulinum, anti-botulinum antibodies, potency, in vitro assay

## Abstract

Botulinum neurotoxins are bacterial proteins that cause botulism, a life-threatening disease. Therapy relies mostly on post-intoxication antibody treatment. The only accepted method to measure the potency of, and to approve, antitoxin preparations is the mouse lethality neutralization bioassay. However, this assay is time-consuming, labor-intensive, costly, and raises ethical issues related to the large numbers of laboratory animals needed. Until now, all efforts to develop an alternative in vitro assay have not provided a valid replacement to the mouse potency assay. In the present study, we report the development of an innovative in vitro assay for determining botulinum antitoxin potency, using botulinum type B as a model. The concept of the assay is to mimic two fundamental steps in botulinum intoxication: receptor binding and catalytic activity. By simulating these steps in vitro we were able to accurately determine the potency of antitoxin preparations. The reproducibility of the assay was high with a CV < 13%. Most importantly, the antitoxin potency measured by the in vitro assay highly correlated with that measured by the standard in vivo mouse assay (*r* = 0.9842, *p* < 0.0001). Thus, this new in vitro assay has the potential to be considered, after validation, as a replacement to the mouse assay for quantitating neutralizing antibody concentrations in pharmaceutical botulinum antitoxin preparations. Future adoption of this in vitro assay would minimize the use of laboratory animals, speed up the time, and reduce the cost of botulinum antitoxin approval.

## 1. Introduction

Botulinum neurotoxins (BoNTs) are bacterial proteins that cause the life-threatening disease botulism, and are considered as among the highest-risk threat agents for bioterrorism (the "Class A agents") [1]. Seven antigenically-distinct BoNT serotypes (designated A to G) are produced by several species of anaerobic *Clostridia*: *C. botulinum, C butyricum, C. baratii*, and *C. argentinense* [2,3,4]. Botulism is a disease with four distinct, naturally-occurring syndromes: foodborne, wound, infant botulism, and adult intestinal toxemia. Inhalational botulism can result from aerosolization of the toxin. All of these result in the same clinical syndrome of symmetrical cranial nerve palsies followed by descending, symmetric, flaccid paralysis of voluntary muscles, which may progress to respiratory compromise and death [5]. An average of 161 cases of botulism occurs annually in the US. Of those, 10% are food-borne, 80% are infant, and 10% are wound botulism [6].

All BoNT serotypes act via similar mechanisms on their target nerve cell [4]: initial binding of the C-terminal portion of the heavy chain through ganglioside and protein receptors on the presynaptic cell surface, followed by internalization into and translocation within the nerve ending by the N-terminal portion of the heavy chain [7]. Inside the nerve terminal, the toxin light chain, which is a zinc-dependent endo-peptidase, cleaves the “soluble N-ethylmaleimide-sensitive factor attachment protein receptor” (SNARE) that promotes fusion and release of acetylcholine [4]. Each BoNT serotype has specific action site. Serotypes A and E cleave the 25 kDa synaptosomal associated protein (SNAP-25), serotypes B, D, F, and G cleave vesicle associated membrane protein (VAMP or synaptobrevin), and serotype C acts on both SNAP-25 and syntaxin [4].

Currently, the only available therapy to botulism patients consists of antibody treatment post-intoxication. In severe cases, mechanical ventilation is also needed. Antitoxin preparations are derived from equine serum mainly due to the availability of large volumes of high potency plasma and to the low-zoonotic character of horses. The clinical benefit from the antitoxin is believed to be the elimination of circulating toxin, which results in reducing the duration and/or severity of the disease [8,9]. Thus, in order to be effective, antitoxin must be administered relatively early in the course of intoxication.

According to the pharmacopeia, the only accepted and standard method to measure the potency of botulinum antitoxin preparations is the traditional mouse lethality neutralization bioassay [10]. In this assay, serial dilutions of an antitoxin are mixed with a constant amount of toxin. The toxin/antitoxin mixtures are incubated in vitro to allow optimal binding and then injected into mice. The potency of the antitoxin is determined by the dose necessary to protect mice against the lethal effect of a test dose of botulinum toxin compared to that of an international standard antitoxin with known potency. However, the mouse assay is time consuming, labor intensive, costly, necessitates a large number of laboratory animals per sample, and takes a long time (up to four days) to complete. Consequently, efforts to develop alternative methods have been made [11,12,13,14,15]. These assays are based on ELISA systems, radio-immune-precipitation assays, mouse hemi-diaphragm, and cell-based assays. However, to date, none of these approaches provide the expected practical benefits over the in vivo mouse assay. Both ELISA and radio-immune-precipitation assays use antibody binding rather than receptor binding and, therefore, do not mimic the natural course of intoxication. Conversely, the mouse hemi-diaphragm and cell-based assays do take into account all phases of intoxication. However, the mouse hemi-diaphragm assay necessitates laboratory animals and can only handle a limited number of samples in a single assay. Similarly, cell-based assays are also limited in the number of tested samples. Moreover, these assays have poor sensitivity and require tedious steps of differentiation prior to conducting the assay. Each BoNT binds to different receptor proteins on presynaptic cell surfaces or to diverse regions of the receptor. For example, BoNT/A enters neurons by binding to the largest luminal loop of the synaptic vesicle protein SV2 (isoforms A, B, and C), with the most robust binding to isoform C. Binding of BoNT/A to a short fragment (amino acids 529–566) within this loop was comparable to binding to the full loop [7]. BoNT/B binds to the synaptotagmin receptor. Frisk et al. found that BoNT/B can bind to amino acids 40–60 of the receptor and showed that binding of a synthetic peptide corresponding to residues 40–60 to magnetic beads enables separation and detection of BoNT/B in micro-channels [16].

One possible reason for the difficulty in developing alternative potency assays could be that some of those assays ignore the catalytic activity of the toxin, which represents the last step of natural intoxication within the nerve terminal. The unique endo-peptidase activity of each neurotoxin serotype has led to the development of in vitro assays for botulinum toxicity in which the biological activity of the neurotoxin itself is used to amplify the assay signal [17,18,19,20]. Briefly, BoNT is incubated with a selective peptide substrate, derived from the relevant BoNT's natural target. The accumulation of BoNT cleavage products is then measured using various detection platforms. These assays have been found suitable for detection of all botulinum serotypes and in many matrices, such as serum, stool, and food [18,21,22,23,24,25,26].

Here we report the development of an alternative in vitro assay for determining the potency of antitoxin preparations. Our concept was to mimic, in vitro, two out of the three major steps in BoNT intoxication: binding to the cellular receptor and catalytic endo-peptidase activity. We chose BoNT/B as a model. For binding, a peptide, representing the binding domain of synaptotagmin, BoNT/B’s cellular receptor, was conjugated to magnetic beads. The toxin binds these peptide-laden-beads with high affinity. Then, an endo-peptidase activity test was conducted with the bound toxin. Initial incubation of the toxin with serial dilutions of antitoxin preparation resulted in dose-dependent activity inhibition. By combining the binding and activity steps, we were able to determine accurately the potency of several antitoxin preparations. The results, obtained using this in vitro assay, were found to be highly correlated with those of the in vivo mouse bioassay. We propose to consider this new in vitro assay, after further validation, as a replacement to the mouse assay. 

## 2. Results

### 2.1. Binding of BoNT/B to Syt-II_40-60_ Peptide

**Figure 1:**
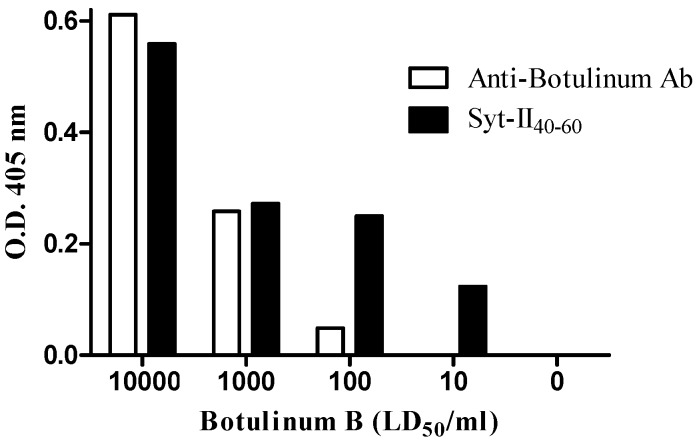
Binding of BoNT/B to Syt-II_40-60_ or to anti-HcB polyclonal antibody-coated magnetic beads by bead-ELISA. Magnetic beads were coated with Syt-II_40-60_ (black bars) or with anti-HcB polyclonal antibodies (open bars) and incubated with 10^1^, 10^2^, 10^3^ and10^4^ MsLD_50_/mL of BoNT/B. Binding was evaluated using colorimetric bead-ELISA assay.

Frisk et al. reported that BoNT/B binds to amino acid 40–60 of the extra-cellular portion of its receptor synaptotagmin [16]. In order to mimic botulinum binding to its receptor, magnetic beads were coated with Syt-II_40-60_ peptide. Control magnetic beads were coated with anti-serotype B-specific polyclonal antibodies. Binding to BoNT/B was evaluated using bead-ELISA. As shown in Figure 1, Syt-II_40-60_-coated beads bind higher amounts of the toxin than did anti-serotype B specific polyclonal antibody-coated beads. The detection limit of Syt-II_40-60_-coated beads was 10 MsLD_50_/mL compared to 10^2^ MsLD_50_/mL with the antibody coated-beads. Thus, we concluded that the toxin binding capacity of Syt-II_40-60_-bound beads is at least as effective as anti-serotype B-specific polyclonal antibody-coated beads.

#### 2.1.1. Application of Bio-Layer Interferometry (BLI) for the Analysis of BoNT/B-Syt-II_40-60_ Interaction

**Figure 2:**
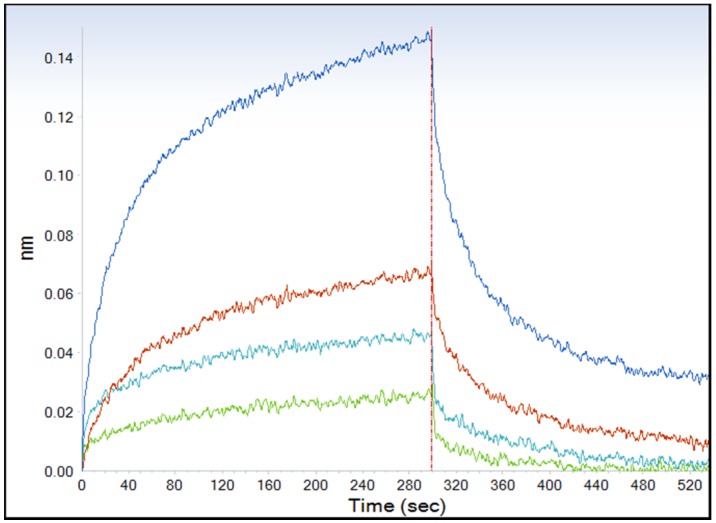
Dose-response curve for binding of BoNT/B to Syt-II_40-60_ biosensor. Various concentrations of BoNT/B were tested by the BLI analysis. Blue, 6 nM (2 × 10^5^ MsLD_50_/mL); red, 3 nM (1 × 10^5^ MsLD_50_/mL); light blue, 1.5 nM (5 × 10^4^ MsLD_50_/mL); and green, 0.75 nM (2.5 × 10^4^ MsLD_50_/mL).

Bio-layer interferometry (BLI) is a label-free technology that can be used for kinetic characterization of protein-protein interactions. To study the kinetic of BoNT/B-Syt-II_40-60_ interaction, SytII_40-60_ was fixed as a solid phase in the BLI system, and its binding to BoNT/B in the running phase was evaluated. The interaction was found to be concentration dependent with kinetics that fits the 2:1 heterogeneous ligand model. This model exhibits two steps binding and dissociation: the first step is rapid (KD = 90 nM) and the second is slow (KD = 20 nM, Figure 2). These results indicate that Syt-II_40-60_ peptide binds BoNT/B with high affinity. The affinity constants measured are in line with other recent reports describing the affinities between BoNT/B and longer forms of Syt-II receptor [27,28].

To test whether toxin that is bound to the synthetic receptor fragment was catalytically active, Syt-II_40-60_-coated beads were allowed to bind BoNT/B and were then incubated with a peptide substrate that contains the specific amino acid sequence that is cleaved by BoNT/B. Quantitation of the cleavage products was conducted following UPLC separation by UV absorbance. Anti-serotype B specific polyclonal antibody-coated beads, that were previously shown to allow measurement of BoNT/B activity in vitro, served as a control [29].

**Figure 3:**
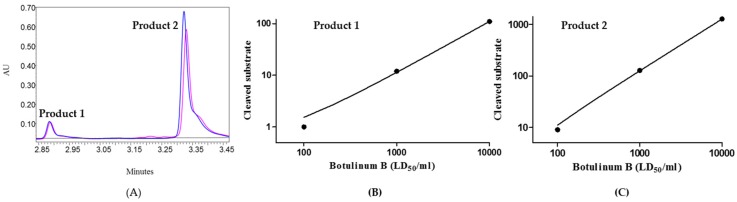
Activity of the bound toxin. (**A**) Ultra Performance Liquid Chromatography (UPLC) chromatograms of cleaved products after extraction with either Syt-II_40-60_ (blue) or anti-HcB polyclonal antibodies (pink) and endo-peptidase reaction. A 10^4^ amount of MsLD_50_/mL BoNT/B were incubated with magnetic beads that were coated with either Syt-II_40-60_ or anti-HcB polyclonal antibodies, then an endo-peptidase reaction was conducted. The cleavage products are shown as two separate peaks in the UPLC chromatogram (at retention times of 2.9 and 3.3 min); (**B**) and (**C**) Syt-II_40-60_-coated beads were incubated with 10^2^, 10^3^, and 10^4^ MsLD_50_/mL BoNT/B. Toxin activity was evaluated by the ability of the bound toxin to cleave a synthetic peptide substrate to its two products (product 1, (**B**), product 2, (**C**)). Product formation was directly proportional to BoNT/B concentration.

BoNT/B captured by either the Syt-II_40-60_ or the polyclonal antibody-based capturing systems, generated comparable amounts of substrate cleavage products (Figure 3A). Incubation of Syt-II_40-60_ coated beads with increased concentration of BoNT/B (10^2^, 10^3^ and 10^4^ MsLD_50_/mL), revealed a linear profile of the cleaved substrate products (*r^2^* = 0.9999 and 1 for the two products, Figure 3B,C, respectively).

### 2.2. Establishment of an in Vitro Anti-BoNT/B Potency Assay

Having shown that Syt-II-bound toxin is catalytically active, we next tested whether BoNT/B specific antitoxin also manifests neutralizing activity in our in vitro system.

**Figure 4:**
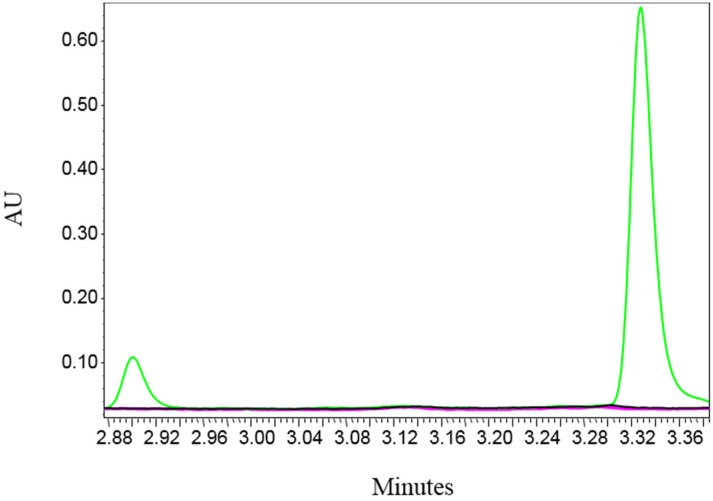
Proof of concept for the in vitro neutralizing assay. Three samples, BoNT/B 10^4^ MsLD_50_/mL alone (green), BoNT/B 10^4^ MsLD_50_/mL with 1 IU standard antitoxin (pink), and control sample without toxin (black), were extracted with Syt-II_40-60_ magnetic beads. After washing, an endo-peptidase reaction was performed. The result was analyzed by UPLC. While BoNT/B generated two cleaved products, addition of the neutralizing antibodies completely abolished toxin activity.

According to the World Health Organization (WHO), 1 IU of antitoxin neutralizes at least 10^4^ MsLD_50_ of toxin [30]. Thus, for a proof of concept, the ability of 1 IU standard BoNT/B antitoxin to neutralize 10^4^ MsLD_50_ BoNT/B in the in vitro assay was tested. For that purpose, three samples were analyzed: 10^4^ MsLD_50_, 10^4^ MsLD_50_ + 1 IU standard antitoxin, and a control sample without toxin. As can be seen in Figure 4, 1 IU standard antitoxin completely abolished the in vitro activity of 10^4^ MsLD_50_ and the cleaved products were undetectable (pink line). When 1 IU standard antitoxin of BoNT/A was used, no inhibition effect was observed (data not shown).

**Figure 5:**
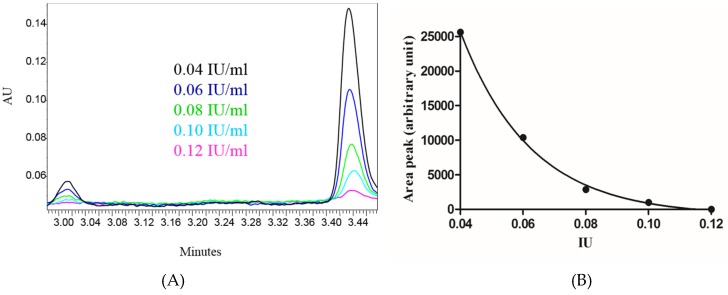
In vitro activity of BoNT/B in the presence of antitoxin standard. (**A**) UPLC chromatograms of BoNT/B cleavage products representing the residual BoNT/B activity generated after incubation of a toxin test dose (2200 MsLD_50_/mL) with the indicated concentrations of a standard antitoxin (0.04–0.12 IU/mL); (**B**) Residual toxin activity for each neutralizing antibody concentration. Activity is presented as the area peak of one of the two cleaved products (3.44 min retention time).

To enable quantification of the potency of unknown antitoxin preparations, a calibration curve with an international standard antitoxin was constructed. For that purpose, we incubated toxin test dose (see Materials and Methods) with several dilutions of a standard antitoxin preparation. After incubation and washing, an endo-peptidase reaction was performed. The area under the UPLC peaks of the subsequent cleavage products was calculated. An example of such UPLC chromatograms is shown in Figure 5A. As can be clearly seen, there is a reverse correlation between the cleaved product peak area and the antitoxin concentration. Analysis of the cleavage products area peaks versus standard antitoxin potencies reveals an exponential decrease (Figure 5B).

### 2.3. Evaluating the Performance of the in Vitro Assay in Quantitating Neutralizing Antibody Concentration in Unknown Antitoxin Preparations

To evaluate the newly developed in vitro assay for potency determination, different antitoxin preparations, which had previously been measured in the standard mouse assay for their potency, were tested in the in vitro neutralization assay using an antitoxin calibration curve. Notably, the tested preparations were both of the intact IgG (I) and F(ab')2 fragments (F). All antitoxin preparations were produced at the IIBR and each sample was originated from a different batch. As seen in Table 1, the results obtained in the in vitro assay had highly reproducibility. The correlation between the anti-BoNT/B neutralizing potencies obtained by the in vitro assay and the gold standard in vivo mouse assay was found to be high and significant (*r* = 0.9909, *p* = 0.0001). As can be seen, the potency of F(ab')2 samples is approximately 3–4-fold higher than that of IgG samples. This difference in potency stems from the nature of the preparation process, and together with the low variability of the assay resulted in potency values that are concentrated in two relatively narrow ranges (~4000–5000 and ~800–1300 IU/mL for F(ab')2 and intact IgG, respectively). 

**Figure 6:**
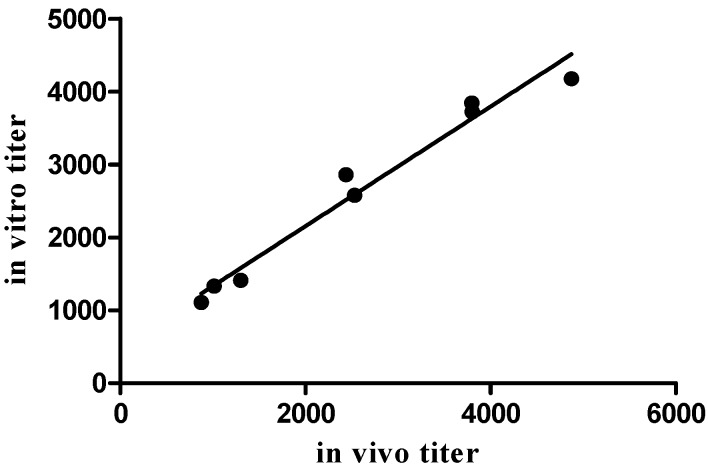
Correlation between antitoxin potencies determined by the in vitro assay and the mouse lethality neutralization bioassay in different antitoxin preparations. The correlation was found to be high and significant, with *r* = 0.9842 and *p* < 0.0001.

In order to further understand the relations between the in vitro and the in vivo potency assays, two spiked samples were prepared by dilution of two independent F(ab')2 samples. The potency of the newly-formed antitoxin samples confirmed the linearity of the in vitro assay, and further extended the correlation between the two assays (*r* = 0.9842, *p* < 0.0001, Figure 6).

## 3. Discussion

Botulism is a toxin-mediated acute neurological disorder that causes potentially life-threatening neuro-paralysis. The only approved therapy for botulism is antitoxin that primarily blocks and neutralizes the circulating neurotoxin. Antitoxin preparations are solutions of sterile products, produced from plasma of healthy horses that have been hyper-immunized against botulinum toxin. These products contain antibody fragments (F(ab')2 portions) obtained by pepsin digestion of the immune plasma, a process that removes the Fc part of the antibody molecule, resulting in significantly reduced risk of sensitization and allergic reactions. The remaining F(ab')2 fragment of the antibody molecule retains most of the neutralizing activity [31]. The antibody fragments are then purified by filtration and chromatographic processes, aimed at removing pepsin and plasma proteins.

The potency of neutralizing antibodies in antitoxin preparations is exclusively measured using a pharmacopeia-approved assay, the mouse neutralization test [32]. The antitoxin potency in the preparation is expressed relative to that of an international standard antitoxin. Although this assay is the only accepted method, it has many disadvantages. For that reason, many research groups are pursuing alternatives. However, all such alternative assays continue to suffer from many drawbacks. ELISA and radio-immune-precipitation assays measure only a single biological property of the antitoxin, binding of the antibody to the toxin. Therefore these methods cannot distinguish between neutralizing and non-neutralizing antibodies. The mouse hemi-diaphragm assay is related as an ex vivo method, but also necessitates consumption of large numbers of laboratory animals. Several cell-based assays have been developed, including ones relying on continuous cell lines, as well as primary neurons [15,33,34]. Theoretically, these assays have the potential for optimal mimicking the toxin natural intoxication and, therefore, were expected to correlate with the mouse bioassay. However, the use of cellular assays for the evaluation of antitoxin potency has not been reported as an alternative for the neutralization mouse bioassay. Reasons may include the need to induce differentiation in most of the cell systems prior to their use in the assay, a step that may interfere with repeatability, and a limitation in the number of antitoxin samples that can be tested in a single assay. Moreover, continuous cell lines exhibit low BoNT sensitivity [35]. Ideally, the potency assay should be able to measure broad range of potencies. As shown in the current research, the new in vitro assay can quantify high levels of antibodies. However, in order to measure antibodies raised in patients that are treated with botulinum and became resistant, it should be able to test low antibodies concentrations. Indeed, preliminary results obtained using the novel in vitro assay show a proof of concept for detection of neutralizing antibodies in human-derived sera (data not shown). Although some primary neuron cell preparations exhibit higher sensitivity, they have poor repeatability and also require laboratory animals for cell preparation. Taken together, despite all these efforts, until now, none of the alternative methods described in the scientific literature were found suitable to replace the standard mouse assay for measuring the potency of neutralizing antibodies in antitoxin preparations.

In this paper we have described the development of a practical in vitro potency assay for anti-BoNT pharmaceutical antitoxin preparations. The concept of this assay is based on in vitro simulation of two out of the three main steps in the natural intoxication process: (1) binding to receptor and (2) the endo-peptidase catalytic activity. Theoretically, an in vitro neutralization assay could have been based on the well-established Endopep assay, which relies on immune-magnetic capturing of the toxin with polyclonal antibodies. The antibodies used to capture the toxin in this assay are specific for the toxin heavy chain, in order to maintain the light chain available for catalytic activity. Most of the neutralizing antibodies in antitoxin preparations are specific for the receptor binding domain, which is located on the toxin heavy chain as well. Hence, competition between capture antibodies and the analyte (neutralizing antibodies) is expected to abolish the ability to measure neutralizing antibodies.

To simulate the binding step, we used a peptide, SytII_40-60_, which represents the binding domain of the toxin receptor, and conjugated it to magnetic beads. The binding domain of the receptor was previously elucidated by Chia et al. using structural data [27]. In the current study, this peptide was conjugated through Streptavidin-Biotin bound in the same orientation of the natural receptor. We found that BoNT/B specifically binds to SytII_40-60_, which is the smallest fragment representing the binding domain of BoNT/B cellular receptor. Examination of the kinetic constant of BoNT/B-SytII_40-60_ binding, not reported to date, revealed that it is in the same range as the kinetic constants described in the literature for larger fragments of the receptor: Brunger and his group found Kd = 34 × 10^−9^ M for binding of HcB (residues 858–1291) with Syt-II_8-61_ conjugated to GST by isothermal titration calorimetry (ITC) [28]. An affinity constant of 2.99 × 10^−7^ M was reported for peptides, derived from amino acid residues H1241-H1277 of BoNT/B, with the luminal domain of Syt-II_37-86_ [36]. Based on the kinetic results, the Kd value was found to be in the ~10–100 nM range, it seems that the short peptide Syt-II_40-60_ maintains the binding characteristics reported for longer portion of this receptor and, therefore, successfully represents the binding domain of the receptor synaptotagmin.

The catalytic step of intoxication was performed with the well-established Endopep assay [17,18,19,20]. The peptide-bound toxin retained its endo-peptidase activity and its cleaved substrate products could be analyzed using UV absorbance. Since botulinum toxin activity in this assay correlates with binding of the toxin to the peptide, it was expected that the addition of neutralizing antitoxin will interfere with its binding ability to the Syt-II_40-60_ peptide. Indeed, the reduced binding led to reduced activity, which then could be ascribed to antibody potency. 

The amino acid sequence of synaptotagmin that was used in this study, ^40^GESQEDMFAKLKEK**F**FNEINK^60^, was derived from the mouse sequence. This decision was based on the need to replace the in vivo mouse assay. This sequence differs from that in humans. When compared to the human sequence, there is a phenylalanine to leucine mutation (F54L, bold and underlined in the sequence above). This mutation eliminates one of three major interactions between synaptotagmin and BoNT/B and is considered to be the reason for the reduced potency of BoNT/B in humans compared to mice. Moreover, this is probably an important basis for difficulties in extrapolating results of animal experiments to pharmaceutical applications in human [37]. The same in vitro assay described in this paper can readily be implemented using the human sequence of the receptor and should theoretically better predict results in human.

We tested several different batches of antitoxin preparations, both of the intact IgG and F(ab')2 fragments, for their potency using this in vitro assay and found high and significant correlation with the standard in vivo mouse assay (*r* = 0.9842, *p* < 0.0001). The reproducibility of the in vitro assay was high with a CV of less than 13%. The high and significant correlation suggests that the in vitro neutralizing assay has a potential to be considered, after further validation, as a replacement to the standard mouse assay in quantitating the concentration of neutralizing antibodies in different pharmaceutical preparations. Future adopting this in vitro assay will minimize the need for laboratory animals and reduce the time and cost of the currently used mouse assay. In addition, this in vitro assay will improve the precision and accuracy of antitoxin potency determination in pharmaceutical preparations, as the CV of the mouse assay, based on a broader number of samples, is about 30%. Implementation of this assay will be relatively easy, attainable, and affordable for any clinical center/agency. Moreover, the concept underlying this novel assay as demonstrated here could be applied to other botulinum serotypes upon elucidation of the relevant binding site on their receptor.

## 4. Materials and Methods 

### 4.1. Ethics Statement

All animal experiments were performed in strict accordance with the Israeli Law and were approved by the Ethics Committee for animal experiments at the Israel Institute for Biological Research (permit no: M-61-2013, approval date: 20/11/2013) all efforts were made to minimize suffering.

### 4.2. Materials

#### 4.2.1. Peptides

Amino acid residues 40–60 of the mouse synaptotagmin protein (^40^GESQEDMFAKLKEKFFNEINK^60^) and residues 60–94 of the VAMP protein (^60^LSELDDRADALQAGASQFETSAAKLKRKYWWKNLK^94^) were synthesized by Proimmune (The Magdalen Centre, Oxford, UK). The synaptotagmin peptide, referred to below as Syt-II_40-60_, was synthesized with a biotin molecule conjugated to lysine at the C-terminus (K-bio). The lyophilized peptides were dissolved in water and their concentrations were determined by UV absorbance using a peptide standard mixture (Sigma-Aldrich Israel Ltd., Rehovot, Israel). Unless stated otherwise, all reagents were from Sigma.

#### 4.2.2. Toxins and Antitoxins

*Clostridium botulinum* strain B was obtained from the IIBR collection (B592). The neurotoxin gene of this strain complies with that of the Danish strain (accession number M81186) [38]. BoNT/B was prepared from concentrated supernatant of a culture grown for six days in anaerobic culture tubes.

Rabbit anti-H_C_/B polyclonal antibodies were purified from the sera of hyperimmune rabbits that were immunized with H_C_/B, as previously described [39]. 

### 4.3. Peptide Conjugation to Magnetic Beads

The Syt-II_40-60_ peptide was bound to Streptavidin magnetic beads. 200 µL of beads (M-280 Dynabeads^®^, Invitrogen, Waltham, MA, USA) were washed twice with 200 µL of washing/blocking buffer (PBS, 1% BSA, 0.025% tween). After washing, the beads were blocked with 200 µL of the same buffer. The beads were then incubated while shaking with 200 µL of washing buffer containing 20 µg Syt-II_40-60_ peptide for 30 min, 37 °C, 800 rpm. The Syt-II_40-60_-conjugated beads were washed three times with 200 µL washing buffer.

### 4.4. In Vivo Mouse Neutralization Antibody Test

The reference method L+10 from the pharmacopeia [40] was used to determine the neutralizing activity of anti-BoNT/B antitoxins preparations. Briefly, serial 1.2-fold dilutions of an antitoxin preparation were prepared. Simultaneously, a standard antitoxin preparation was diluted to concentrations of 0.08, 0.10, 0.12, 0.14 International Units per mL (IU/mL). Then, all antitoxin dilutions were incubated for one hour at 25 °C with a toxin test dose of 2200 MsLD_50_ previously determined according to the pharmacopoeia. Each mixture was injected I.P. into four mice (1 mL per mouse), and survival was monitored for four days. Antitoxin potency was calculated based on the lowest dilution of antitoxin that failed to protect the animals, compared to that of the standard antitoxin.

### 4.5. In Vitro Neutralization Assay 

Dilutions of the toxin and the antitoxins were identical to those described in the in vivo assay section. Additional standard antitoxin dilutions used were 0.04 and 0.06 IU/mL. All antitoxin dilutions were incubated for one hour at 25 °C with a toxin test dose of 2200 MsLD_50_. Then, each toxin/antitoxin mixture (1 mL) was incubated while shaking with 40 µL Syt-II_40-60_-conjugated magnetic beads for one hour, 37 °C, 800 rpm. Next, the supernatant was removed and the beads were washed three times with decreasing volumes (1, 0.5, 0.2 mL) of PBS containing 0.1% BSA and 0.025% tween, followed by three additional washing steps with 0.2 mL PBS (after the second wash with PBS, the beads were transferred to a new tube). After the final washing step, the supernatant was removed.

### 4.6. Endo-Peptidase Activity Assay

Endo-peptidase activity was measured as described previously [18,29,41,42,43]. Briefly, the reaction was performed in a 20 µL reaction volume containing toxin-bound beads, 100 µM peptide substrate, 1 mM ZnCl_2_, 1% Triton, 10 mM dithiothreitol (DTT), and 50 mM HEPES buffer (pH 7.3) at 37 °C with shaking for five hours. 180 µL of 1% formic acid was added and the samples were incubated for 2 min at 100 °C. Beads were removed prior to UPLC analysis.

### 4.7. UPLC Analysis

A 10 µL aliquot of each reaction supernatant was analyzed with Waters Acquity UPLC (Waters Corporation, Milford, MA, USA) equipped with a UV detector and binary solvent manager. The output signal was monitored and processed using Empower software. The method was developed using an Acquity UPLC BEH C18 1.7 µm (2.1 × 50 mm) column. The flow rate of the mobile phase was 0.15 mL/min. The column temperature was 50 °C and the eluted products were monitored at a wavelength of 215 nm. The cleaved products were rinsed for 3 minutes in an acetonitrile gradient from 95% buffer A (5% acetonitrile in 0.1% TFA) and 5% buffer B (80% acetonitrile in 0.1% TFA) to 70% buffer A. Quantitative analysis of the products was performed by calculating the area of the product's peak using a standard calibration curve. Unless stated otherwise, all UPLC equipment were from Waters and materials from Sigma.

### 4.8. Bead ELISA

Fifty microliters of magnetic beads coated with either anti-HcB polyclonal antibody or Syt-II peptide were blocked with blocking buffer. After discarding the supernatant, 200 µL of buffer was added to each tube, and the tubes were split into five different aliquots (each containing 40 µL). For four out of five pairs of tubes (one containing anti-HcB antibodies coated beads and the other containing peptide coated beads), 200 µL of PBS containing toxin (10^1^, 10^2^, 10^3^, 10^4^ mouse MsLD_50_/mL) was added. The fifth pair served as a control and contained 200 µL of buffer. The tubes were incubated for two hours at 37 °C, 800 rpm, washed three times and 200 µL of polyclonal anti-BoNT/B antibody diluted 1:4000 was added. Next, beads were incubated for 1 hour at 37 °C and washed again. Goat anti-horse (200 µL, 1:4000) was added to each tube for one hour at 37 °C, which were centrifuged at 800 rpm and washed again, four times. After washing, 30 µL Sure-Blue solution was added and the reaction was incubated at 37 °C until color developed. The reaction was stopped with 60 µL of 0.5 M H_2_SO_4_, then transferred to a transparent plate and read in 450 nm with an Infinite 200 plate reader (Tecan, Mannedorf, Switzerland).

### 4.9. Biolayer Interferometry

Biolayer interferometry (BLI) experiments were conducted using an Octet Red instrument (ForteBio, Menlo Park, CA, USA). The Syt-II_40-60_ peptide, representing the sequence of the BoNT/B binding site on Syt-II, was immobilized on a Streptavidin tip by incubating the sensor in 0.2 mL peptide solution (4 pg/mL) for two minutes. Assays were performed in a solid black 96-well micro-plate at 25 °C. Measurement cycles consisted of three steps: (1) equilibration in buffer (PBS + 0.02% Tween 20; 1% BSA; 0.05% azide) for two minutes to establish a baseline signal; (2) association with toxin for two minutes; and (3) dissociation in buffer. After each cycle the tip was regenerated by incubation in regeneration buffer (50 mM glycine-HCl pH = 2.7) for 15 seconds. Four concentrations of toxin were prepared: 6, 3, 1.5, and 0.75 nM, (2 × 10^5^, 1 × 10^5^, 5 × 10^4^, and 2.5 × 10^4^ MsLD_50_/mL, respectively). Data analysis was done using ForteBio Data Analysis 7.0 (ForteBio, Menlo Park, CA, USA) for determination of constants (KD = Koff/Kon, where KD = equilibrium dissociation constant, Kon = association rate constant, and Koff = dissociation rate constant).

## Figures and Tables

**Table 1:** Determination of anti-BoNT/B neutralizing antibodies potencies in different antitoxin preparations using our in vitro assay and the in vivo mouse lethality neutralization bioassay.

**Preparation**	**Anti-BoNT/B Antibody Potencies**					
	**Mouse Assay (IU/mL)**			**Endopep Assay (IU/mL)**		
	**Average (*n*)**	**SD**	**CV (%)**	**Average (*n*)**	**SD**	**CV (%)**
F-1	4875 (3)	650	13	4178 (4)	171	4.1
F-2	3798 (3)	605	16	3843 (3)	469	12.2
F-3	3800 (3)	0	0	3722 (3)	294	7.9
F-1/2 *	2438 **	NA	NA	2860 (3)	250	8.7
F-3/1.5 *	2533 **	NA	NA	2576 (3)	290	11.3
I-1	1330 (1)	NA	NA	1403 (3)	54	3.8
I-2	1014 (1)	NA	NA	1330 (3)	143	10.8
I-3	874 (1)	NA	NA	1105 (3)	96	8.7

* Dilution factor from the original factor, ** Calculated.
